# Emulation of X-ray Light-Field Cameras

**DOI:** 10.3390/jimaging6120138

**Published:** 2020-12-11

**Authors:** Nicola Viganò, Felix Lucka, Ombeline de La Rochefoucauld, Sophia Bethany Coban, Robert van Liere, Marta Fajardo, Philippe Zeitoun, Kees Joost Batenburg

**Affiliations:** 1 ESRF—The European Synchrotron, 38043 Grenoble, France; 2 Centrum Wiskunde & Informatica (CWI), NWO, 1098 XG Amsterdam, The Netherlands; Felix.Lucka@cwi.nl (F.L.); s.b.coban@cwi.nl (S.B.C.); Robert.van.Liere@cwi.nl (R.v.L.); K.J.Batenburg@cwi.nl (K.J.B.); 3 Centre for Medical Image Computing, University College London, London WC1V 6LJ, UK; 4 Imagine Optic, 33400 Talence, France; odlrochefoucauld@imagine-optic.com; 5 Mathematics and Computer Science, Technical University Eindhoven, 5600 MB Eindhoven, The Netherlands; 6 Instituto de Plasmas e Fusão Nuclear, Instituto Superior Técnico, Universidade de Lisboa, 1049-001 Lisboa, Portugal; marta.fajardo@tecnico.ulisboa.pt; 7 Laboratoire d’Optique Appliquée, ENSTA, CNRS, Institut Polytechnique Paris, 91762 Palaiseau, France; philippe.zeitoun@ensta-paris.fr; 8 Leiden Institute of Advanced Computer Science, Leiden University, 2333 CA Leiden, The Netherlands

**Keywords:** X-ray, plenoptic imaging, light-field, single-shot 3D imaging

## Abstract

X-ray plenoptic cameras acquire multi-view X-ray transmission images in a single exposure (light-field). Their development is challenging: designs have appeared only recently, and they are still affected by important limitations. Concurrently, the lack of available real X-ray light-field data hinders dedicated algorithmic development. Here, we present a physical emulation setup for rapidly exploring the parameter space of both existing and conceptual camera designs. This will assist and accelerate the design of X-ray plenoptic imaging solutions, and provide a tool for generating unlimited real X-ray plenoptic data. We also demonstrate that X-ray light-fields allow for reconstructing sharp spatial structures in three-dimensions (3D) from single-shot data.

## 1. Introduction

**Figure 1:**
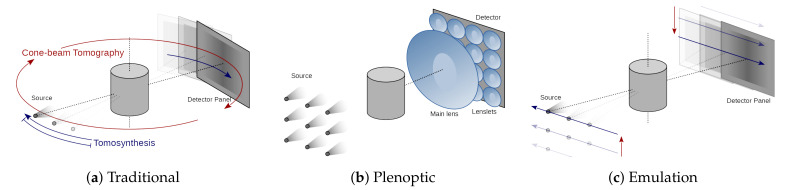
(**a**) Traditional cone-beam computerized tomography (CBCT) and linear trajectory tomosynthesis acquisition geometries; (**b**) plenoptic camera design; and, (**c**) emulated plenoptic acquisition using a flexible X-ray scanner.

X-ray imaging is ubiquitous as a tool for medicine, industry, and science. While conventional radiography is commonly used for simple medical examinations and high-throughput industrial quality inspection, it lacks the three-dimensional (3D) information that is required for a thorough assessment of the internal state of the scanned object. By combining a series of X-ray images computationally, a 3D volumetric representation of the object can be reconstructed that provides a much higher level of detail. This concept is extensively used in medical computerized tomography (CT) scanners, but also in tomosynthesis [1], laminography, and a variety of other 3D X-ray imaging techniques. However, all of these techniques are based on sequential acquisition of multiple X-ray images, thereby imposing strong constraints on the speed and dose by which a full 3D scan can be carried out (Figure 1a). In fact, while CT has been one of the greatest achievements in 3D X-ray imaging, it comes with relatively long scanning times and high dose levels.

Plenoptic imaging is a technique that is demonstrated in the visible regime, and that employs an array of micro-lenses and a pixelized detector in order to obtain a single-shot photograph. This special photograph allows for computational refocusing after acquisition [2]. The micro-lenses sort the incoming light into different spatial bins at the detector, depending on their incidence angle (Figure 1b). In [3], it was shown that a light-field image is geometrically equivalent to a limited-angle cone-beam CT (CBCT) acquisition, establishing a bridge between the geometrical concepts of plenoptic visible-light photography and CT imaging (Figure 1c).

**Figure 2:**
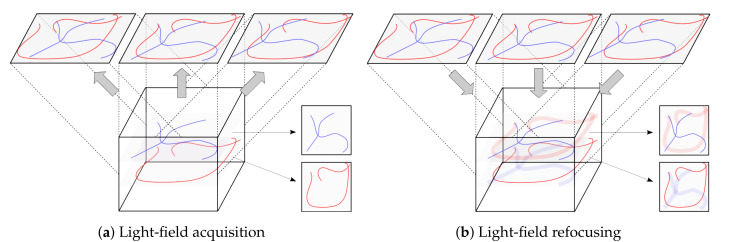
Sketch of traditional light-field operations: (**a**) light-field acquisition from the illuminated volume; and, (**b**) integration refocusing (shift-and-add) of the focal stack. Sharp in-plane structures are superimposed with blurry out-of-plane structures.

An X-ray plenoptic camera acquires multi-view X-ray transmission images in a single exposure. This allows for bringing several superimposed 3D structures into focus at their respective depth planes, instead of only the object surface, as in visible-light imaging. If the structures are located at a particular depth and have sharp features, focusing on them enables determining their depth and segmenting their shape (Figure 2). This effectively allows for fast depth-resolved 3D imaging that is only limited by the flux of the X-ray source and framerate of the detector.

The equivalence between the plenoptic acquisition and limited-angle CBCT [3] allows us to use iterative CT algorithms for the image reconstruction [4]. Furthermore, it shows that X-ray light-field images offer a bi-dimensional out-of-plane acquisition (Figure 1c): the sample projections are not acquired along a linear trajectory, as in tomosynthesis (Figure 1a), which only allows for detecting features that are oriented parallel to the detector or in the transverse direction with respect to the acquisition trajectory. In contrast, the projections are acquired on a plane that is parallel to the detector, which allows for detecting arbitrarily oriented features.

**Figure 3:**
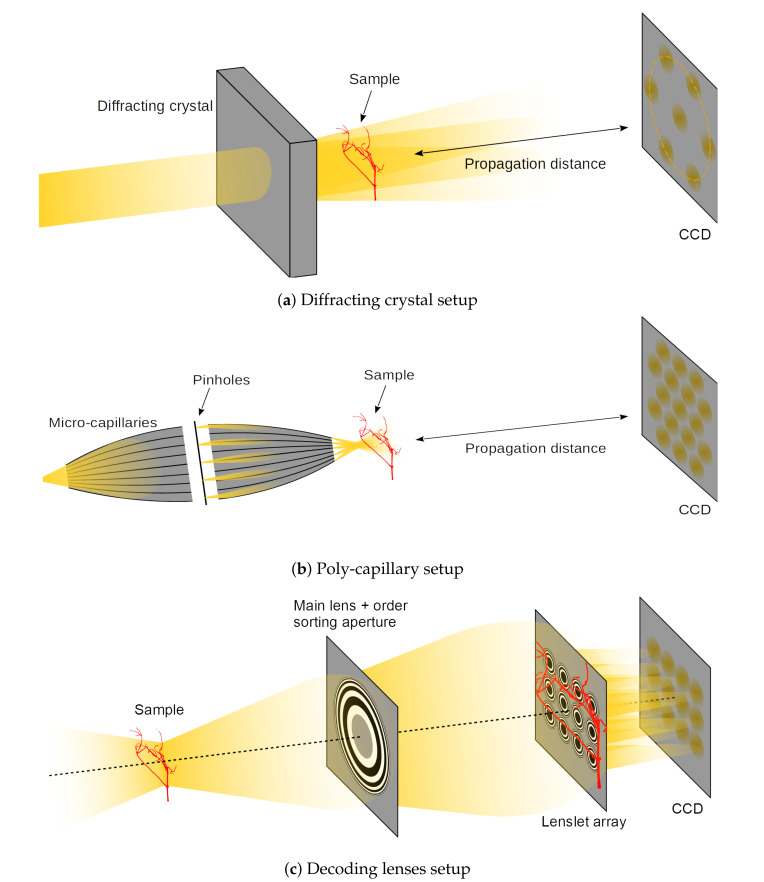
Considered X-ray plenoptic apparatuses: (**a**) diffracting crystal based multi-projection design; (**b**) poly-capillary based multi-projection design; and, (**c**) lenses-based X-ray plenoptic camera design.

Despite its appealing features, X-ray plenoptic imaging has only seen limited development so far. Two apparatuses that are capable of producing X-ray light-fields have been developed: one uses a relatively large crystalline object and a monochromatic parallel-beam [5] (Figure 3a) and the other one uses poly-capillary optics and a poly-chromatic cone-beam [6] (Figure 3b). While these apparatuses represent ingenious solutions for achieving X-ray plenoptic images, they suffer from a few limitations. Both of them are fixed in the fabrication configuration, and they do not allow adjustments for different samples, field-of-view (FoV), angular sampling, or angular range. Moreover, the acquisition can only be considered to be plenoptic in a significantly small region of space around the focal plane (e.g., for the micro-capillary solution in [6], a region of ±6μm around the focal plane in the depth direction). On top of that, the former has a fixed relationship between the angular sampling parameters (e.g., sampling range) and incoming beam energy for any given crystal structure, which, in turn, constrains the type or thickness of the samples for a given depth resolution. The latter suffers from significantly low flux delivered to the sample, which results in exposures of 3 min. per image, as shown by the same authors in previous publications with the same setup [7]. This might render the implemented plenoptic imaging setup not competitive against traditional CT setups.

The just described apparatuses share the design choice of having optics before (upstream) the sample. These optics structure the illumination in beamlets, which shine through the sample from different directions. Subsequently, they rely on the propagation of the diverging beamlets to separate (demultiplexing) the different sub-aperture images. This approach presents the advantage of being dose efficient, because no absorbing optics are placed after (downstream) the sample, and virtually all of the transmitted photons can be captured. In visible plenoptic imaging, the reverse approach is used: the sample is illuminated with diffuse illumination and the decoding optics are placed downstream of the sample (Figure 3c). Despite being potentially less dose efficient than the upstream optics plenoptic imaging (UOPI) counterparts, these downstream optics plenoptic imaging (DOPI) apparatuses have the advantage of offering greater flexibility: they allow for adapting the focusing distance and angular sampling range on-the-fly, by simply changing the distances between the different optical elements. In this article, we do not advocate for any specific design out of these different choices. Instead, we develop a physical acquisition scheme that allows for geometrically emulating all of them, and to acquire the corresponding X-ray light-fields.

The just described complexity of creating X-ray plenoptic imaging apparatuses has hindered the development of the field. The scarcity and problems of the currently available apparatuses has, in turn, made it difficult (if not impossible) to get hold of X-ray light-fields. Thus, no algorithm has been developed so far to specifically take advantage of the appealing features of this single-shot data, while dealing with its limitations and challenges (e.g., semi-transparency, as seen in Section 3). Our emulation scheme allows for reproducing the X-ray light-field acquired by a hypothetical X-ray plenoptic camera, before even purchasing the optics. The single-shot acquisition is mechanically emulated with a flexible X-ray laboratory scanner by moving the source to sequential positions, without the need for X-ray lenses (Figure 1c). This new tool will assist and markedly accelerate the design and development of new X-ray plenoptic imaging solutions, by allowing for quickly testing a wide variety of them, with different parameters, in a cost efficient manner (once the flexible scanner is available). Equally importantly, it will provide access to unlimited X-ray light-fields of widely different resolutions and angular sampling ranges, which will unlock the development and interest for dedicated algorithmic development. This is the X-ray equivalent to the light-field acquisition gantry setups from Stanford [8], which allowed for acquiring iconic visible light datasets that are still used as benchmarks nowadays. These datasets gave the visible plenoptic imaging community a head start for the development of the refocusing and depth-estimation algorithms, when it was difficult to obtain a plenoptic imaging setup. The only main limitation of our approach is that the emulation is only valid for monochromatic illumination, except for the poly-capillary case.

In Section 2, we describe the UOPI and DOPI apparatuses in detail, and provide the emulation procedure for each of them. In Section 3, we demonstrate the ability of the proposed scheme to emulate and predict the performance of the DOPI designs. In Section 4, we discuss the following three aspects: relationship between X-ray plenoptic imaging and the already existing stereo and tomographic imaging, the impact of this work on the design of both UOPI and DOPI apparatuses, and the problems that are related to the lack of existing X-ray plenoptic data and specific processing algorithms. Finally, in Section 5, we conclude the article by outlining a few examples of research fields and applications that could benefit from X-ray plenoptic imaging.

## 2. Method

The proposed emulation scheme for acquiring X-ray light-fields is a procedure that uses a flexible X-ray laboratory scanner, and it can be adjusted in order to emulate both the UOPI and DOPI configurations. This procedure requires movements of the X-ray source (XRS) and flat panel sensor (FPS) with respect to the sample in both the horizontal and vertical directions (as sketched in Figure 1c). The position of the XRS and FPX are mapped in the (u,v) space, where *u* and *v* are the horizontal and vertical transverse coordinates to the optical axis of the system. A pin-hole image (PHI), which is also known as sub-aperture image (SAI), is taken at specific points in the (u,v) coordinates, which are identified as S(u,v) and D(u,v) for the XRS and the center of the FPS, respectively. If we trace lines from all of the points in the S(u,v) to the corresponding points in the D(u,v) set, then they cross in one specific point along the optical axis, which corresponds to the position of the focal plane of the imaging system. The XRS-to-focus distance is called $z_{0}$, while the XRS-to-FPS distance is called $z_{sd}$.

While all of the described setups work in the hard X-ray regime, the energy ranges differ from setup to setup. The working ranges are indicated in each section of the different apparatuses.

### 2.1. Large Diffracting Crystal

**Figure 4:**
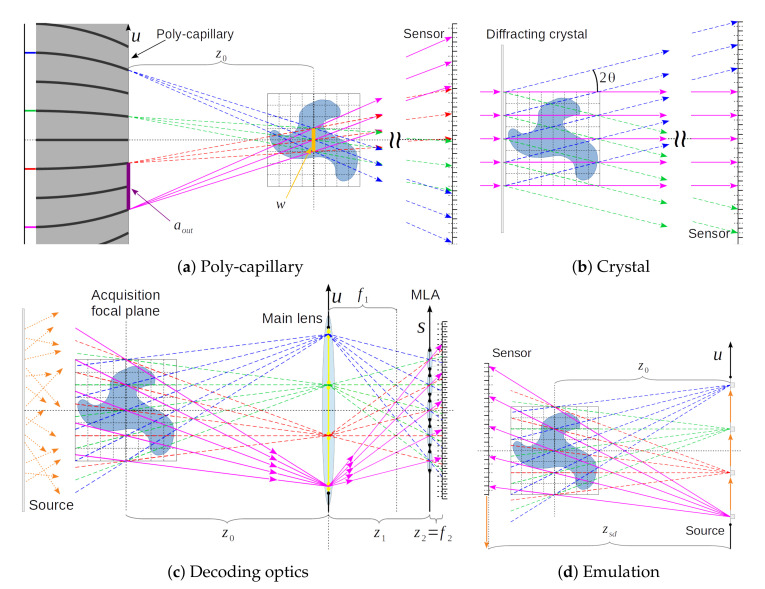
X-ray plenoptic acquisition schemes: (**a**) poly-capillary acquisition scheme; (**b**) diffracting crystal acquisition scheme; (**c**) decoding lenses acquisition scheme (with diffusive source); and, (**d**) tomographic emulation apparatus.

The plenoptic apparatus based on a diffracting crystal is presented in Figure 3a, and schematically sketched in Figure 4b. A two-dimensional monochromatic hard X-ray parallel beam shines through a diffracting crystal, which produces a transmitted beam and multiple Bragg reflections. The transmitted and scattered beams overlap in a region that is close to the crystal due to the extended size of the incoming beam. The sample is placed in this overlap region. The FPS is placed downstream the sample, where the diverging beams are not overlapping. The diffracted beams are usually much less intense than the transmitted beam. The angles between the incident beam and the diffracted beams will change, depending on the X-ray beam energy. For more details we refer to [5], where the authors used an incoming beam energy of 4 keV.

The plane of the diffracting crystal corresponds to the focal plane of the plenoptic imaging system, and each beam on the FPS produces a different pin-hole image (PHI) of the sample. The PHI center positions P(u,v) are determined by the reciprocal lattice of the diffracting crystal structure and, for their determination, we refer to specialized textbooks, like [9] or [10]. In our emulation scheme:

(1) D(u,v)=P(u,v),

(2) $$S(u,v)=-\frac{z_{0}}{z_{sd}-z_{0}}P\left(u,v\right)\;.$$

This configuration assumes a parallel beam, but the XRS of our emulation setup produces a conic beam. This produces a size and depth distortion in the acquired light-field, which can be computed and corrected, as discussed in [3]. However, within the limits of the travel ranges of the XRS, it is possible to reduce this effect by having $\left(z_{sd}-z_{0}\right)\ll z_{0}$.

### 2.2. Poly-Capillary Elements

The plenoptic apparatus based on poly-capillaries is presented in Figure 3b and schematically sketched in Figure 4a. A poly-chromatic X-ray conic beam from a laboratory XRS shines through a first poly-capillary element, which renders the beam approximately parallel. The resulting parallel X-ray beam is then filtered though a multi-pin-hole mask that selects a sparse array of beamlets, which are then focused with a second poly-capillary element. The focal plane of the optical system is where all of the beamlets overlap. The FoV of the region is equal to the beamlet waist. Past the focal plane, the beamlets diverge and separate. A FPS is placed downstream the sample, where all of the beamlets do not overlap any more and form PHIs.

The energy dispersion of the poly-capillary elements’ response presents itself as a vignetting effect, where increasing energies are transmitted less and less at higher angles. This is due to the poly-capillary material having a total-reflection critical angle that decreases with increasing energy [7]. This effect and the intensity profile of X-ray lab sources both contribute to higher photon counts for the central PHIs with respect to the peripheral PHIs in the recorded light-field.

The pin-hole mask is placed close to the second poly-capillary entry, and it defines the arrangement of the PHIs on the FPS. The beamlet source positions can be approximately positioned at the exit point of the second poly-capillary element. Given an entrance distance of $a_{in}$ and exit distance $a_{out}$ between neighbouring poly-capillaries, the taper factor $t=a_{out}/a_{in}$ defines the demagnification factor for the pin-hole mask given by the poly-capillary. If ϕ is the critical angle for total external reflection of the material that is used for the poly-capillary elements (for borsilicate glass ϕ[mrad]≈30 / E [keV]), the beamlet waist *w* is equal to $2z_{0}\phi$ at the focal position $z_{0}$. This means that the following inequality has to be satisfied, in order to have separation of the beamlets through propagation: $a_{out}>w$, imposing an inverse relationship between the angular resolution and the FoV and this apparatus. For more details, we refer to [6,7], where the authors used a X-ray tube with a mean energy of 9 keV.

In our emulation scheme, the XRS positions coincide with the beamlet source positions, the distances $z_{0}$ is equal to the focal distance of the poly-capillary element, and the FPS is positioned at the same position of the FPS in the emulated apparatus. Given a certain hole distribution H(u,v) of the pin-hole mask:

(3) $$H\left(u,v\right)=p_{u}\;\delta u\;\hat{u}+p_{v}\;\delta v\hat{v}\;,$$

where $\hat{u}$ and $\hat{v}$ are the unit vectors of the *u* and *v* axes, respectively, and $p_{u}$ and $p_{v}$ are integers. The resulting XRS and FPS positions are:

(4) S(u,v)=tH(u,v)

(5) $$P(u,v)=-\frac{z_{sd}-z_{0}}{z_{0}}\;tH\left(u,v\right)\;.$$

### 2.3. Decoding Lenses

The plenoptic apparatus based on decoding lenses is presented in Figure 3c and schematically sketched in Figure 4c. It is a conceptual design, and it is based on the acquisition scheme of a visible-light plenoptic camera (Figure 1b), but it employs sophisticated diffractive X-ray optics.

This apparatus is composed of a main lens (ML), a micro-lenses array (MLA), and a FPS (Figure 4c). Let $f_{1}$ and $f_{2}$ be the focal distances of the ML and MLA, respectively, while $z_{1}$ and $z_{2}$ the distances ML-to-MLA, and MLA-to-sensor, respectively. The distance between the ML and acquisition focal plane is $z_{0}$. Here, we limit ourselves to the unfocused plenoptic geometry for simplicity [2,11,12]. This implies $z_{2}=f_{2}$. The coordinates (u,v) identify positions on the ML’s plane, (s,t) on the MLA’s plane, and (σ,τ) on the portion of sensor behind every lenslet. The spatial resolution of the acquired images (δs,δt) is equal to the physical size of one micro-lens (Δs,Δt) divided by the ML magnification $\left|M\right|=z_{1}/z_{0}$, and the angular resolution (δu,δv) on the ML equal to the sensor pixel-size (Δσ,Δτ) divided by the micro-lens magnification $\left|m\right|=z_{2}/z_{1}$ [3]. The set of pixels at the same corresponding position (σ,τ) under every lenslet receives intensity from the same position (u,v) on the ML, and it forms a PHI. The spacing between adjacent PHIs on the *u* axis is δu, and so the numerical aperture (NA) of the ML determines the maximum number of PHIs recorded. We define the angular range of the PHIs as the arctangent of twice the maximum (u,v) coordinate, divided by the distance $z_{0}$. The XRS is supposed to illuminate the sample with diffused light. For this purpose, either the XRS should have a focal spot larger than the sample size or the setup should use an X-ray diffuser. In both cases, the generated light is expected to be incoherent.

For simplicity, we assume that this type of apparatuses can be built whlile using diffractive X-ray optics, in the form of Fresnel zone plates (FZPs). We purposely ignore efficiency and fabrication aspects of the FZPs (e.g., feature size and aspect ratio), because they pertain to technical development aspects that are beyond the scope of this article. However, we recognise that the ML is the most critical component from a fabrication stand point: the required NAs may not be achievable with a single FZP with the current fabrication technologies. Alternative implementations include near field stacking of the zone-plates with mismatched feature position [13], or pairs of multilayer Laue lenses could be used [14]. The former could also be a solution for improving the efficiency of FZPs [15], in view of the requirements for dose-sensitive or time-critical applications. Additionally, for simplicity, we assumed that only the first-order of the FZPs would be present, while, in a real case, the zeroth-order as well as higher positive and negative orders would be present. Using an order sorting aperture might not be ideal in this case, because it would restrict the accepted angular range of each lens. Thankfully, in practice, all of these other orders will be out-of-focus and will not interfere because of the incoherent illumination. This will give rise to low frequency signals that can be treated as background, and most likely subtracted during pre-processing. The efficiency and focusing distance of FZPs depend on the incoming beam energy. For practical reasons, we expect this setup to work best with energies up to 20 keV.

For the emulation of this design, we use the findings of [3]: the line integrals through the sample space are propagation direction independent, and so they can be reversed. Thus, the position of the pin-hole images on the main-lens of the DOPI design correspond to the X-ray source positions in the emulation scheme:

(6) $$S\left(u,v\right)=p_{u}\;\delta u\;\hat{u}+p_{v}\;\delta v\hat{v}\;,$$

where $\hat{u}$ and $\hat{v}$ are the unit vectors of the *u* and *v* axes, respectively, and $p_{u}$ and $p_{v}$ are integers. The resulting similar geometry can be clearly seen by comparing Figure 4c against Figure 4d. Given the XRS-to-FPS distance $z_{sd}$ and position P(u,v) of the X-ray source (XRS), the detector (FPS) is then placed at the opposite side of the sample with respect to the XRS, at positions:

(7) $$D\left(u,v\right)=-\frac{z_{sd}-z_{0}}{z_{0}}\;S\left(u,v\right).$$

The lines that connect the XRS to a given pixel (x,y) on the FPS always cross the same point on the plane at the distance $z_{0}$ for every matching couple of positions P(u,v) and D(u,v). The emulated spatial resolution (δs,δt) is equal to the FPS resolution (δx,δy) divided by the cone-beam magnification $z_{sd}/z_{0}$. This acquisition scheme allows for emulating a wide variety of plenoptic cameras using only a limited number of parameters from the emulated camera: the resolutions (δs,δt) and (δu,δv), the number of PHIs allowed by the ML’s NA (maximum $p_{u}$ and $p_{v}$), and the distance $z_{0}$.

## 3. Experiments

Here, we report on experiments that were carried out with the custom-built *FleX-ray* CT scanner at CWI [16], which provides automated source and detector positioning. For these experiments, we demonstrate the ability of the proposed scheme to emulate and predict the performance of the conceptual design of Section 2.3. This serves as an example of its use both as a setup design tool, and for producing the desired X-ray light-field data. The data that are used in this section have been published on *zenodo*, and referenced in the relevant sub-sections.

### 3.1. Experimental Parameters

The emulated DOPI setup is composed of: a Fresnel zone plate (FZP) used as ML and placed 49.48 mm downstream to the sample, a MLA of 128×128 FZPs at 243.71 mm from the ML, and a FPS at 8.78 mm from the MLA. The ML has a 1000μm diameter and 3 nm last zone width. The micro-lenses have a 32μm diameter, 36μm pitch, and 20 nm last zone size. The resulting angular range of the PHIs is $1.029^{{}^{\circ}}$. The emulated source is point-like and monochromatic, with an energy of 17 keV. The FPS pixel size is 4μm. Various choices for the implementation of the optics can be made, as this is just an example of a possible design, which results in a family of camera designs.

A sufficiently large NA is necessary for meeting the required angular range (≈1${}^{{}^{\circ}}$) and resolution of plenoptic acquisitions. In our emulated example, this translates to NAs of at least 0.01 (≈0.6${}^{{}^{\circ}}$) for the ML and 0.0016 (≈0.09${}^{{}^{\circ}}$) for the micro-lenses. The choice for FZPs imposes lower bounds on the required lens NA and efficiency. To date, we are not aware of the successful fabrication of FZPs, like the one that is needed in the example for the ML. As discussed in Section 2.3, this could change either through technical breakthroughs is the FZP fabrication process, or the implementation of clever strategies, like zone-plate stacking, or alternatively through the use of pairs of multilayer Laue lenses in place of the FZPs.

In the experiments that are reported here, the scanner FPS pixel size is 150μm, the source-to-sample distance, set equal to $z_{0}$, is 890.725 mm, and the source-to-FPS distance $z_{sd}$ is 1012.95 mm, resulting in an effective acquisition pixel size Δs, Δt of ≃131.55μm. The 128×128 pixel PHIs have been acquired on a 9×9P(u,v) grid, with a spacing of 2 mm between neighbouring points. The XRS is a modified Spellman XRF source, being limited to 90 kV maximum voltage and 90 W maximum power output, with a 20μm focal spot size.

This scanner setup emulates a magnified version of the camera in the spatial dimensions by a factor of 18. It is important to notice that the angular range of the emulation is the same as the one of the reference setup.

### 3.2. Line-Like Features

**Figure 5:**
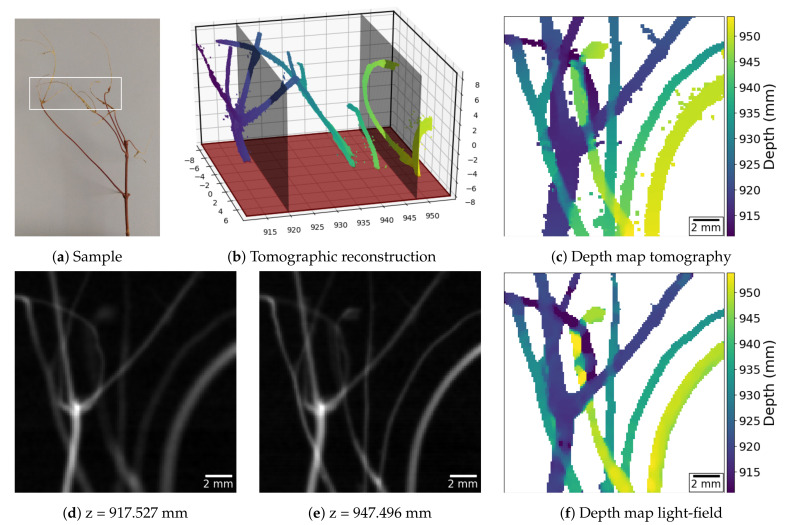
X-ray light-field imaging of a tree branch to demonstrate the ability to capture the three-dimensional (3D) structure of vessel-like objects. The sample is shown in (**a**), where the white box identifies the analyzed region. The traditional CBCT reconstruction is rendered in (**b**). (**d**,**e**) present the refocus planes shown in (**b**). (**c**,**f**) present the depth maps extracted from the tomographic reconstruction and the light-field acquisition, respectively.

For the first experiment, we imaged a branch (Figure 5a), whose segments present thicknesses that range from 0.1 mm to 1.2 mm. Taking the 18 times magnification into account, this corresponds to using the reference X-ray plenoptic camera to image vessels with thicknesses between 5.55μm and 66.66μm, which is comparable to blood vessels from the size of capillaries up to the size of arterioles. The raw and pre-processed light-field data as well as the raw tomographic acquisition data with its reconstruction and segmentation volumes, for this example, are available on *zenodo* at [17].

A ground truth depth map was obtained through traditional CBCT (Figure 5b), and it is shown in Figure 5c. The depth map computed from the acquired light-field is shown in Figure 5f. The two maps look remarkably similar in both the morphology and depth of the reconstructed objects, despite the light-field acquisition angular range of ∼1${}^{{}^{\circ}}$, and the depth-estimation being performed without an explicit model of the underlying vessel structures. In contrast, stereo imaging systems can also be used for positioning sharp structures in 3D space, but their matching mechanism requires larger angular ranges, relatively simpler structures, and no superposition, as recently shown in [18].

The highest depth-estimation error is usually associated with segments of the sample, which, despite being completely disjoint in the depth direction, do occupy adjacent regions in the central view of the light-field. The reason for this effect is the current limitation in the data analysis algorithms, which do not handle the overlap of semi-transparent objects in the PHIs.

### 3.3. Sphere-Like Features

**Figure 6:**
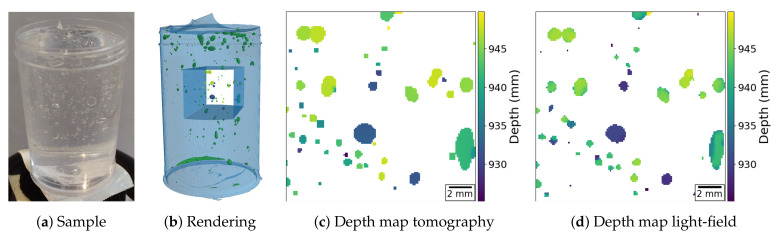
X-ray light-field imaging of air bubbles in gel to demonstrate the ability to capture the three-dimensional (3D) position and shape of sphere-like objects. (**a**) Sample; (**b**) Rendering of the tomographic reconstruction with emphasis on analyzed region; and, (**c**,**d**) present the depth maps extracted from the tomographic and the light-field reconstruction, respectively.

A second experiment with the same acquisition geometry is presented in Figure 6. Here, we demonstrate the ability of a plenoptic X-ray image to determine the position and size of sphere-like objects by imaging a tube filled with hair gel and air bubbles. The results that are presented in Figure 6d show that the particle positions and sizes are in good agreement with the ground truth in Figure 6c (obtained as in the previous example). The raw and pre-processed light-field data as well as the raw tomographic acquisition data with its reconstruction and segmentation volumes, for this example, are available on *zenodo* at [19].

**Figure 7:**
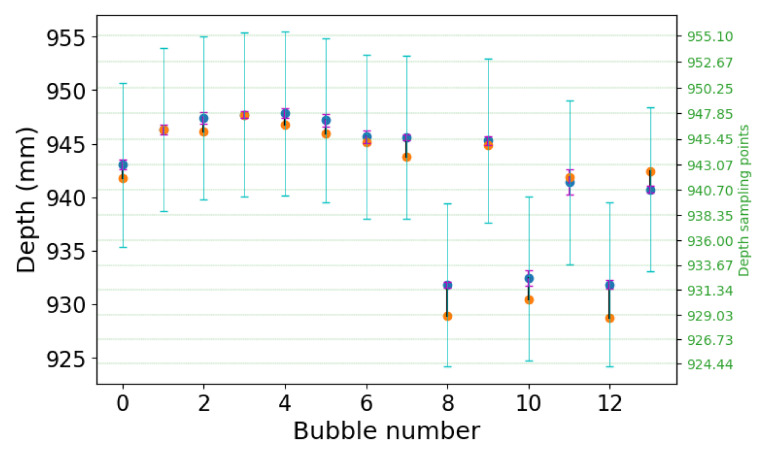
Performance evaluation of the depth-estimation of the light-field acquisition (Figure 6d) against the tomographic acquisition (Figure 6c). For the 13 largest bubbles, the blue dots indicate the expected depth for the center of the bubbles, while the orange dots indicate the reconstructed depth. The magenta bars indicate the bubble size, and the cyan bars indicate the expected uncertainty from the limited angle. The green horizontal dashed lines indicate the depths sampling used for the creation of the focal stack. The corresponding depths are indicated on the right axis.

Figure 7 shows a quantification of the depth-estimation performance. We selected the 13 largest bubbles from Figure 6, and compared their computed average depth from the light-field data against the expected average depth from the tomographic reconstruction. The deviation is also compared against the expected uncertainty on the measurement, when considering the ∼1${}^{{}^{\circ}}$ acquisition angular range of the light-field acquisition. The blue dots indicate the expected depth of the bubbles, while the orange dots indicate the reconstructed depth. The magenta bars indicate the bubble size, and the cyan bars indicate the expected measurement uncertainty. The reconstructed depths are well within the uncertainty regions, which confirms the striking similarity that is observed in Figure 6.

The depth uncertainty size is equivalent to the depth resolution of a tomographic reconstruction with the same angular range. In plenoptic imaging, this is also known as the depth-of-field (DoF) of the refocused images, and it is presented in [3]. For this example, the DoF is equal to 15.05 mm, which results in an uncertainty of 7.65 mm around the expected value. The reconstructed depths have a mean error of 1.27 mm with a standard deviation of 0.94 mm, which is much smaller than the expected uncertainty.

### 3.4. Data Processing

**Figure 8:**
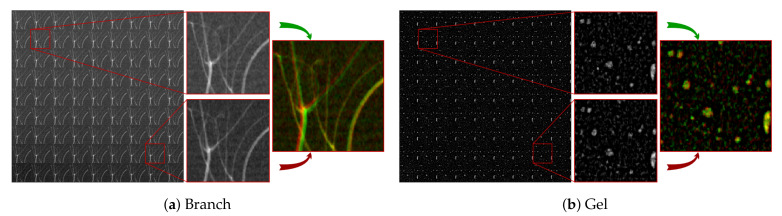
Tiled pin-hole images (PHI) of the two analyzed datasets: (**a**) small branch; (**b**) hair gel with air bubbles. Both of the figures include the magnification of two different PHIs. The two PHIs are then combined into an RGB image, in order to better show their differences. The top image contributes to the green channel, and the bottom image to the red channel.

Figure 8 shows the X-ray data that were used for the presented examples, in the form of tiled PHIs. In both Figure 8a,b, two PHIs are also magnified, and they are composed into an RGB figure to better show their differences.

Concerning the pre-processing of the data, the light-fields have been normalized while using open-beam images with corresponding XRS and FPS positions for each PHI. After normalization, the PHIs in Figure 8b have been additionally background-subtracted, while using blurred versions of themselves. The used rectangular filter was 21 pixels in both the *s* and *t* dimensions. This operation has removed the curved container influence from the projections. Following background subtraction, the contrast of the PHIs in Figure 8b has been inverted, in order to give the bubbles positive signal.

Slight errors in the shape of the objects can occur in the light-field depth-maps, due to the empty background. The depth estimation algorithm always assumes having a recognizable pattern across the whole picture. Thus, it is not able to distinguish the imaged objects from the background. In order to reduce this problem, we have applied a mask to the depth-map, which was computed by segmenting the sum of the focal stack along the depth direction.

Concerning the depth-maps, the algorithm from [20] was used for both X-ray light-fields. This algorithm assumes that only one object is visible in the line of sight of one pixel. Thus, in Figure 5f, the algorithm returned an average of the distances of two branches when they crossed. A similar approach was taken for the tomography depth-maps (Figure 5c and Figure 6c), where the reported distance in each pixel is the average of the distances of all the objects on the said pixel’s line of sight. The depth-estimation algorithm uses two cues for guessing depth: defocus and correspondence. The former looks at the strongest high-frequency signal throughout the focal stack for each pixel in the map. The latter looks at the smallest deviation between the refocused images and the acquired PHIs throughout the focal stack for each pixel in the map. The two cues are then merged together while using a regularized convex optimization procedure. The regularization imposes some degree of sparsity in the gradient and laplacian space of the depth-map. For more details, we refer to [20].

The software package that was used for the depth estimation has been developed by the authors, and released as open source under the GPL-3.0 license. The package is called *plenoptomos*, and it can be found on *github* at [21].

## 4. Discussion

These experiments show that depth and 3D position of sharp substructures can be computed from light-field data. When combined with the single-shot aspect of X-ray plenoptic apparatuses, this will allow for depth-resolved 3D imaging at high framerates, which is competitive against both tomographic and stereo imaging approaches. Stereo imaging systems have considerable limitations and constraints when compared to plenoptic imaging systems, as discussed in Section 3.2: they need larger angular ranges and relatively simpler structures, and they prefer an explicit model of the underlying structures, as recently shown in [18]. Instead, when compared to the current tomographic methods, which allow only framerates of up to 4.5 Hz [22], X-ray plenoptic imaging systems can offer acquisition speeds that are comparable to stereo imaging. In fact, the X-ray plenoptic acquisition is only limited by the flux of the X-ray source, the efficiency of the optics, and framerate of the detector, while tomographic systems rely on mechanical movements around the sample.

X-ray plenoptic imaging is currently limited by technological aspects in both the apparatus realization and data processing. The current UOPI designs have fixed FoV size, as well as spatial and depth resolutions, which are intrinsic to the materials used or the fabrication of the optical elements. This lack of flexibility requires careful planning of the setup in order to fulfill the requirements of the target application. The conceptual DOPI designs, on the other hand, present realization challenges, and they raise potential concerns with the deposited dose on the sample. In fact, due to a limited efficiency of the decoding optics, which are placed after the sample, a higher dose might be needed in order to achieve the required image quality for the required data processing. In both the UOPI and DOPI designs, our emulation scheme allows for evaluating the performance of different setups before fabrication. In the former case, it can support specific design choices of the setup, while, in the latter, it allows for assessing technological requirements for the imaging performance, and also identifying the areas where development efforts are unnecessary. In both cases, it also gives an assessment of the expected 3D imaging performance of the emulated setup.

The data processing software for current generation X-ray light-field data is based on the algorithms from visible light-field data, where semi-transparent objects are either not handled or handled poorly. This produces errors in the depth-estimation reconstruction, as seen in Section 3.2. The lack of specific development is due to the unavailability of X-ray light-fields, as mentioned in the introduction. Our emulation scheme allows for emulating a wide range of designs and parameters, providing access to unlimited X-ray light-field data.

## 5. Conclusions and Outlook

By exploiting the recently established equivalence between the plenoptic imaging geometry and limited-angle CBCT, we presented a method that uses a laboratory X-ray system with moving components in order to physically emulate the X-ray plenoptic camera, apart from a magnification factor. This provides a way to quantify the potential of a camera design and guide its development, even before the actual prototype implementation. The plenoptic system, without moving components, will then be able to reach comparable imaging results for similar structures with single-shot imaging, only limited in time-resolution by the X-ray detection apparatus and XRS flux.

We used this method to image vessel-like and particle-like structures that are encountered in important application areas of X-ray imaging. Our computational results demonstrate that detailed and accurate depth-maps can be reconstructed from the acquired X-ray data that are representative of the prospective plenoptic X-ray data. In a number of clinical and scientific applications, the advantages of X-ray plenoptic imaging are profound and, thus, could lead to breakthroughs, including: (a) imaging of blood vessels based on contrast injection, (b) particle image velocimetry (PIV) for tracking fluid flows in opaque media or containers, and (c) the real-time observation of metallic foams formation or dedritic growth and solidification processes. In particular, for case (a), interventional angiographic imaging requires compact imaging apparatuses and high framerates, which prohibit tomographic approaches, but the current single-projection and stereo-projection approaches lack the depth information for resolving complex vessel networks. Figure 5 demonstrates the advantages of X-ray plenoptic imaging for this scenario. For case (b), plenoptic visible-light PIV is an established technique for its ability to extract the full particle directional and positional information at high framerates. The presented examples tackle structures that are very similar to the ones that were observed in all three cases. This shows that X-ray light-field imaging is well suited for those scenarios.
